# Malnutrition and cachexia among cancer out-patients in Nairobi, Kenya

**DOI:** 10.1017/jns.2017.61

**Published:** 2017-12-28

**Authors:** L. U. Kaduka, Z. N. Bukania, Y. Opanga, R. Mutisya, A. Korir, V. Thuita, C. Nyongesa, M. Mwangi, C. F. L. Mbakaya, E. Muniu

**Affiliations:** 1 Centre for Public Health Research, Kenya Medical Research Institute, Nairobi, Kenya; 2 School of Public Health, Moi University, Eldoret, Kenya; 3 Centre for Clinical Research, Kenya Medical Research Institute, Nairobi, Kenya; 4 Department of Nutrition, Kenyatta National Hospital, Nairobi, Kenya; 5 Texas Cancer Centre, Nairobi, Kenya; 6 Rongo University, Rongo, Kenya

**Keywords:** Malnutrition, Cancer, Body composition, Kenya, Sub-Saharan Africa, AOR, adjusted OR, ESPEN, European Society of Clinical Nutrition and Metabolism, FFMI, fat-free mass index, FVS, Food Variety Score, KEMRI, Kenya Medical Research Institute, KNH, Kenyatta National Hospital, MUST, Malnutrition Universal Screening Tool, TCC, Texas Cancer Centre

## Abstract

Cancer is the third leading cause of death in Kenya. However, there is scarce information on the nutritional status of cancer patients to guide in decision making. The present study sought to assess the risk of malnutrition, and factors associated with malnutrition and cachexia, among cancer out-patients, with the aim of informing nutrition programmes for cancer management in Kenya and beyond. This was a facility-based cross-sectional study performed at Kenyatta National Hospital and Texas Cancer Centre in Nairobi, Kenya. The risk of malnutrition was assessed using the Malnutrition Universal Screening Tool (MUST). Diagnoses of malnutrition and cachexia were done using the European Society of Clinical Nutrition and Metabolism (ESPEN) and Fearon criteria, respectively. A total of 512 participants were assessed. Those at risk of malnutrition were 33·1 % (12·5 % at medium risk, 20·6 % at high risk). Prevalence of malnutrition was 13·4 %. The overall weight loss >5 % over 3 months was 18·2 % and low fat-free mass index was 43·1 %. Prevalence of cachexia was 14·1 % compared with 8·5 % obtained using the local criteria. Only 18·6 % participants had received any form of nutrition services. Age was a predictor of malnutrition and cachexia in addition to site of cancer for malnutrition and cigarette smoking for cachexia. The use of the MUST as a screening tool at the first point of care should be explored. The predictive value of current nutrition assessment tools, and the local diagnostic criteria for malnutrition and cachexia should be reassessed to inform the development of appropriate clinical guidelines and future capacity-building initiatives that will ensure the correct identification of patients at risk for timely care.

Cancer is the third leading cause of death in Kenya with a mortality rate estimated at 26 941 annually^(^^1^^)^. Malnutrition and cachexia are common in cancer and often contribute to poor health outcomes. Malnutrition is a clinical condition characterised by an imbalance of energy, protein and other nutrients that causes measurable adverse effects on body tissue, function and clinical outcome^(^^2^^)^. Cancer cachexia is a multifactorial syndrome characterised by an ongoing loss of skeletal muscle mass, with or without loss of fat mass that cannot be fully reversed by conventional nutrition support^(^^3^^)^. Cachexia is a manifestation in advanced cancer that affects 50–80 % of cancer patients and accounts for up to 20 % of cancer deaths^(^^4^^,^^5^^)^. Early detection and management of malnutrition and cachexia are therefore key. This has necessitated the integration of nutrition care in cancer management for improved clinical outcomes and quality of life^(^^6^^)^.

The 2013 National Guidelines for Cancer Management in Kenya identifies nutrition care for persons with cancer as an integral part of cancer treatment^(^^7^^)^. It singles out the need for early initiation of management plans that incorporate nutritional assessment, diagnosis and intervention. BMI and weight loss are currently the main indicators of malnutrition and cachexia, respectively, in Kenyan public hospitals. However, previous studies have shown the limitations of using BMI as the sole indicator of malnutrition in cancer because it can yield high or normal results in patients with large tumours, ascites or oedema, thereby masking weight loss. Unintentional weight loss is instead recommended^(^^8^^,^^9^^)^.

The Malnutrition Universal Screening Tool (MUST) is designed to identify adults who are underweight or obese and at risk of malnutrition, and who may benefit from appropriate intervention^(^^2^^)^. It uses BMI, weight loss and acute disease effect scores in estimating the overall risk of malnutrition. The European Society of Clinical Nutrition and Metabolism (ESPEN) consensus statement provides a minimum set of criteria for the diagnosis of malnutrition that can be applied independently of clinical setting and aetiology^(^^10^^)^. It allows for inclusion of parameters such as fat-free mass where applicable in diagnosing malnutrition. The international consensus on definition and classification of cancer cachexia also provides diagnostic criteria for cachexia that include weight loss or loss of skeletal muscle mass^(^^3^^)^. These assessments enrich understanding of the variability in nutritional status, and have the potential to alter cancer treatment paradigms for improved health outcomes^(^^11^^,^^12^^)^.

There is scarce information on the burden of malnutrition and cachexia among cancer out-patients in Kenya to inform decision making and policy^(^^1^^)^. In view of the lack of information and the recent advances in screening and diagnosis of malnutrition and cachexia, this study sought to establish the risk of malnutrition, and factors associated with the occurrence of malnutrition and cachexia, with the ultimate aim of informing nutrition programmes for cancer management in Kenya and beyond.

## Research methods

### Study design and population

This was a facility-based cross-sectional study among cancer out-patients seeking treatment at Kenyatta National Hospital (KNH) and Texas Cancer Centre (TCC) in Nairobi, Kenya. KNH is the largest public referral hospital in Kenya. The KNH cancer treatment centre receives more than 22 000 cancer patients annually^(^^13^^)^. TCC, on the other hand, is a private facility that receives approximately 1092 cancer patients annually^(^^14^^)^.

### Inclusion criteria

The study included all out-patients with confirmed stage I–IV cancer disease, aged above 18 years and receiving treatment (radiotherapy, chemotherapy, surgery or hormonal) in the two selected cancer treatment sites.

### Sample size estimation and allocation

Prevalence of malnutrition among cancer patients in Kenya remains unknown. Using the Fischer formula^(^^15^^)^, a sample size of 512 participants was calculated factoring in a 10 % non-response rate. The calculated sample size was distributed between the two facilities using the square root allocation method to ensure appropriate representation of the two facilities based on the number of patients received per facility per year. A systematic random sampling method was used in recruiting participants until the desired sample size was achieved. This entailed obtaining the total number of patients expected in each facility from the hospital records and selecting a random starting point. The sampling interval was determined by dividing the total number expected by the desired sample size.

### Recruitment and training of research assistants

Research assistants with a medical background were recruited and trained on the protocol, study procedures including the consenting process, study tools and on proper data collection using pretested data collection tools. Training and certification were done to harmonise data collection methods between researchers. This was followed by piloting of the study tools and definitive data collection.

### Assessments

#### Sociodemographic assessments

Data collection was done between June and July 2016. Interviews were carried out and information on socio-economic and demographic characteristics collected using a pretested structured questionnaire. Education level was categorised into four categories: none, primary (1–8 years), secondary (9–14 years), and tertiary (>14 years). Occupation was classified as formal, non-formal, unemployed and retirees.

#### Anthropometric assessments

Weight and height were measured using calibrated Seca 762 classic mechanical medical weighing scales and UNICEF standard height boards, and findings recorded to the nearest 0·1 kg and 0·1 cm, respectively. In measuring height, participants were asked to stand upright, without head gear and shoes, arms hanging loosely at the sides, feet slightly apart, and with the back of the heels and head against the stadiometer. In measuring weight, the scale was placed on a flat surface and zero checked before every measurement. Participants were weighed without shoes while wearing minimal clothing. Weight and height measurements were used to compute BMI using the formula of BMI = weight (kg)/height (m^2^). Information on weight history was retrieved from patients’ medical records and used to calculate changes in weight. The current weight was deducted from the previous weight to calculate the amount of weight lost.

#### Body composition assessments

Total body fat, body water and lean mass percentages were estimated using a commercially available single-frequency four-electrode bioimpedance analyser system (Bodystat 1500). Tetrapolar hand to foot measurements were performed in a supine position lasting for 15 min. The gel-filled electrodes were placed on the dorsal surfaces of the right hand and foot, distal ones being, respectively, proximal to the metacarpal and metatarsal phalangeal joints in accordance with standard tetrapolar electrode placement to minimise gap impedence^(^^16^^)^. The instrument recorded whole body impedance from the hands to the feet by applying an electric alternating current flux of 0·8 A at an operating frequency of 50 kHz. Fat-free mass index (FFMI) was calculated using the formula FFMI = lean body mass (kg)/height (m^2^).

#### Clinical and dietary assessments

Details on participants’ diagnosis (clinical history, types of cancer and clinical stages) and treatment were retrieved from patient files. Information on symptoms such as lack of appetite, feeling nauseated or vomiting was self-reported. Dietary diversity was assessed using individual Food Variety Scores (FVS) that collect data on specific food items consumed^(^^17^^)^. Participants provided details on the variety of foods consumed 7 d and 24 h prior to the interview. Each food item was given a score of 1 if consumed at least once over 24 h and the 7 d period regardless of the frequency. The FVS were classified as either adequate by creating a cut-off point where the mean of the upper tercile was used to classify the respondent as either having adequate or inadequate dietary diversity.

#### Risk of malnutrition

Participants were screened using the MUST to identify those at risk of malnutrition. The overall risk was an aggregate of BMI, weight loss and acute disease scores whose cut-off points and interpretation of the data were done in accordance with MUST guidelines^(^^2^^)^. BMI cut-offs of >20, 18·5–20 and <18·5 kg/m^2^ were scored as 0, 1 and 2, respectively. Unplanned weight loss of <5, 5–10 and >10 % in the past 3–6 months was scored as 0, 1 and 2, respectively. The acute disease effect did not apply to the out-patients and was scored a zero. A score aggregate of 0, 1 and 2 represented low, medium and high risk of malnutrition, respectively.

#### Diagnosis of malnutrition

Diagnosis of malnutrition was done in accordance with the ESPEN criteria for the diagnosis of malnutrition^(^^10^^)^. The criteria provide for the use of low BMI (<18·5 kg/m^2^), or combined finding of unintentional weight loss of >5 % over 3 months and at least one of either a reduced BMI of <20 kg/m^2^ or <22 kg/m^2^ in subjects younger and older than 70 years, respectively, or a low FFMI of <15 kg/m^2^ and <17 kg/m^2^ in females and males, respectively, to diagnose malnutrition. Similar to ESPEN, the local criterion uses low BMI in diagnosing malnutrition; hence further bivariate and multivariate analyses were done using low BMI.

#### Diagnosis of cachexia

Diagnosis of cachexia was in accordance with the Fearon criteria for the assessment of cachexia^(^^3^^)^. Diagnosis of cancer cachexia was based on BMI < 20 kg/m^2^ and any degree of weight loss >2 %. The local definition of cachexia is >5 % weight loss/month or >10 % weight loss over 6 months^(^^7^^)^.

Information from the MUST will be used to identify patients at risk of malnutrition for targeted intervention. In addition to information on malnutrition based on low BMI, differences in malnutrition based on weight loss and either a reduced age-specific BMI or low sex-specific FFMI diagnostic criteria will help inform clinical practice, the development of guidelines and appropriate care plans for cancer out-patients in Kenya.

#### Ethical considerations

This study was conducted according to the guidelines laid down in the Declaration of Helsinki and all procedures involving human subjects were approved by the Kenya Medical Research Institute (KEMRI) Scientific and Ethics Review Unit (reference: KEMRI/SERU/CPHR/001/3026) and the KNH Ethics and Research Review Committee (registration certificate: P462/07/2015). Written informed consent was obtained from all study subjects.

#### Data management and statistical analysis

Quantitative data were double-entered using the Microsoft Access application. Clean and validated data were exported to SPSS version 20.0 statistical software (IBM Corp.) for data analysis. Exploratory data analysis techniques were used at the initial stage of analysis to uncover the data distribution characteristics of continuous variables and identify outliers. Descriptive statistics such as proportions and frequency distributions were used to summarise categorical variables and measures of central tendency and dispersion for continuous variables. Pearson's χ^2^ test or Fisher's exact test, where applicable, was used to assess the relationship between dependent and independent categorical variables. To test for association between independent continuous variables and dependent categorical variables, the unpaired Student's *t* test for normally distributed continuous variables and the Mann–Whitney *U* test for non-normally distributed continuous variables were used for dichotomous dependent categorical variable. For dependent categorical variables with more than two categories, one-way ANOVA for normally distributed continuous variables and Kruskal–Wallis one-way ANOVA for non-normally distributed continuous variables were carried out. The threshold for statistical significance was set at α = 0·05 (two-sided). *Post hoc* grouping of study participants was done during analysis since this was a comparative study.

## Results

A total of 512 participants were recruited (male: 28·1 %; female: 71·9 %). The mean age was 52 (sd 13·8) years. The majority of the participants (71·7 %) were aged 40 years and above, 20·3 % were in formal employment, and 9·8 % had not received formal education. The top three site cancers were cancer of the breast (28·7 %), female genital (22·7 %) and digestive organs (21·7 %). The majority (260; 52 %) of the patients presented with late-stage cancer (stages III and IV) compared with 213 (42·6 %) with early-stage cancer (stages 0, I and II). Table 1 shows the sociodemographic and clinical characteristics of the study participants.

**Table 1.** Sociodemographic and clinical characteristics of study participants (*n* 512) (Numbers and percentages)

Characteristics	*n*	%
Sex		
Female	368	71·9
Male	144	28·1
Age (years)		
Mean	52·01	
sd	13·78	
<30 years	22	4·3
30–39 years	72	14·1
40–49 years	133	26·0
50–59 years	135	26·4
60 years and above	150	29·3
Education levels		
No formal education	50	9·8
Primary	195	38·1
Secondary	171	33·4
Tertiary	96	18·8
Occupation		
Formal	104	20·3
Non-formal	198	38·7
Unemployed	170	33·2
Retired	40	7·8
Marital		
Married	378	73·8
Single	75	14·6
Other*	59	11·5
Stage of cancer (*n* 500)		
Stage 0	4	0·8
Stage 1	75	15·0
Stage 2	134	26·8
Stage 3	141	28·2
Stage 4	119	23·8
Unknown	27	5·4
Site of cancer		
Breast (C50)	147	28·7
Female genital (C51–58)	116	22·7
Digestive organs (C15–26)	111	21·7
Lip, oral cavity and pharynx (C00–14)	94	18·4
Haematopoietic (C81–96)	9	1·8
Bone, cartilage, melanoma (C40–43)	18	3·5
Respiratory organs (C30–39)	9	1·8
Kaposi sarcoma (C46)	8	1·6

* Divorced, widowed or separated.

Breast (39·7 %) and cervical (25 %) cancer were the leading cancers in females, and prostate (24 %) and oesophagus (24 %) in males. Up to 470 (91·8 %) participants were on treatment, out of whom 202 (43 %) were on one form of treatment (chemotherapy 114 (24·3 %), surgery sixteen (3·4 %), radiotherapy sixty-eight (14·5 %) and hormonal therapy four (0·8 %)), while 183 (38·9 %) and eighty-five (18·1 %) were on two and three combined forms of treatments, respectively. Fatigue and poor appetite were reportedly the most common symptoms experienced in the previous 24 h (38·3 and 32·4 %), and 1 month at 50 and 51 %, respectively. Up to 35 % reported feeling nauseated while 28 % had vomited in the previous 1 month. Only 18·6 % of the participants reported having received nutrition services in the form of nutrition counselling and education or food support during their hospital visits.

### Risk of malnutrition

The risk of malnutrition was assessed in 471 participants. Those found at risk of malnutrition were 33·1 % (12·5 % at medium risk and 20·6 % at high risk). The risk of malnutrition was significantly higher in males (OR 2·84 (95 % CI 1·87, 4·31); *P* < 0·001), patients who smoked cigarettes (OR 2·86 (95 % CI 1·76, 4·66); *P* < 0·001), those with stage II cancer disease (OR 1·97 (95 % CI 1·12, 3·43); *P* = 0·017), and those on radiotherapy treatment (OR 2·35 (95 % CI 1·36, 4·06); *P* = 0·002), as shown in Table 2. Those aged <30 years were less likely to be at risk (OR 0·26 (95 % CI 0·09, 0·67); *P* < 0·001), and so were patients with breast cancer (OR 0·34 (95 % CI 0·18, 0·63); *P* = 0·001).

**Table 2.** Sociodemographic and clinical factors associated with risk of malnutrition (Numbers and percentages; odds ratios and 95 % confidence intervals)

	At risk*		Not at risk*				
Characteristic	*n*	%	*n*	%	OR	95 % CI	*P*
Overall	156	33·1	315	66·9			
Sex							
Female	89	57·1	249	79·0	Reference		
Male	67	42·9	66	21·0	2·84	1·87, 4·31	<0·001
Age							
<30 years	13	8·3	8	2·5	0·26	0·09, 0·67	0·006
30–39 years	25	16·0	44	14·0	0·74	0·39, 1·37	0·335
40–49 years	35	22·4	88	27·9	1·05	0·61, 1·81	0·848
50–59 years	44	28·2	82	26·0	0·78	0·46, 1·31	0·356
60 years and above	39	25·0	93	29·5	Reference		
Cigarette smoking							
Yes	44	28·2	38	12·1	2·86	1·76, 4·66	<0·001
No	112	71·8	277	87·9	Reference		
Alcohol consumption							
Yes	61	39·1	95	30·2	1·49	1·00, 2·22	0·053
No	95	60·9	220	69·8	Reference		
Stage of cancer							
Unknown	12	7·9	13	4·2	0·71	0·29, 1·70	0·444
Stage 0	0	0	3	1·0	UD	UD	
Stage 1	26	17·2	43	13·9	1·08	0·58, 2·01	0·793
Stage 2	31	20·5	93	30·1	1·97	1·12, 3·43	0·017
Stage 3	38	25·2	90	29·1	1·56	0·91, 2·66	0·107
Stage 4	44	29·1	67	21·7	Reference		
Site of cancer							
Lip, oral cavity and pharynx (C00–14)	33	21·2	54	17·1	Reference		
Digestive organs (C15–26)	46	29·5	53	16·8	1·42	0·79, 2·55	0·241
Respiratory organs (C30–39)	3	1·9	4	1·3	1·22	0·25, 5·83	0·797
Bone, cartilage, melanoma (C40–43)	9	5·8	7	2·2	2·10	0·72, 6·18	0·176
Breast (C50)	24	15·4	116	36·8	0·34	0·18, 0·63	0·001
Female genital (C51–58)	35	22·4	73	23·2	0·78	0·43, 1·42	0·421
Haematopoietic (C81–96)	5	3·2	3	1·0	2·72	0·61, 12·17	0·189
Kaposi sarcoma (C46)	1	0·6	5	1·6	0·32	0·03, 2·93	0·318
Type of treatment							
Chemotherapy	95	68·8	238	82·1	Reference		
Surgery	10	7·2	17	5·9	1·47	0·65, 3·33	0·352
Radiotherapy	31	22·5	33	11·4	2·35	1·36, 4·06	0·002
Hormonal therapy	1	0·3	2	1·4	5·01	0·45, 55·91	0·19

UD, undetermined; MUST, Malnutrition Universal Screening Tool.

* Risk of malnutrition assessed using the MUST ((BMI cut-offs of >20, 18·5–20 and <18·5 kg/m^2^ representing low, medium and high risk, respectively) + (unplanned weight loss of <5 %, 5–10 % and >10 % in the past 3–6 months representing low, medium and high risk, respectively)).

### Prevalence and factors associated with malnutrition

Malnutrition was assessed in 506 participants. Malnutrition based on BMI < 18·5 kg/m^2^ was 13·4 % (males, 24·8 %; females, 8·9 %). Bivariate analysis revealed males to be 3·39 (95 % CI 2·01, 5·73) times likely to be malnourished compared with females. Likewise, the odds of having malnutrition were higher among cigarette smokers (3·19 (95 % CI 1·81, 5·60); *P* < 0·001), alcohol consumers (2·01 (95 % CI 1·20, 3·38), *P* = 0·008), patients with stage II cancer (2·30 (95 % CI 1·02, 5·17); *P* = 0·044), those with cancer in digestive organs (2·56 (95 % CI 1·22, 5·35); *P* = 0·012), and those undergoing surgery (3·26 (95 % CI 1·35, 7·86); *P* = 0·002) and radiotherapy treatment (2·37 (95 % CI 1·19, 4·69); *P* = 0·002). The opposite was observed in patients less than 30 years of age and those with breast cancer disease, as shown in Table 3.

**Table 3.** Sociodemographic and clinical factors associated with malnutrition (Numbers and percentages; odds ratios and 95 % confidence intervals)

	With malnutrition*		Without malnutrition*				
Characteristic	*n*	%	*n*	%	OR	95 % CI	*P*
Overall	68	13·4	438	86·6			
Sex							
Female	32	47·1	329	75·1	Reference		
Male	36	52·9	109	24·9	3·39	2·01, 5·73	<0·001
Age							
<30 years	8	11·8	14	3·2	0·23	0·08, 0·63	0·004
30–39 years	9	13·2	62	14·2	0·90	0·38, 2·14	0·812
40–49 years	20	29·4	112	25·6	0·73	0·37, 1·47	0·379
50–59 years	14	20·6	120	27·4	1·12	0·53, 2·37	0·765
60 years and above	17	25·0	130	29·4	Reference		
Cigarette smoking							
Yes	24	35·3	64	14·6	3·19	1·81, 5·60	<0·001
No	44	64·7	374	85·4	Reference		
Alcohol consumption							
Yes	32	47·1	134	30·6	2·01	1·20, 3·38	0·008
No	36	52·9	304	69·4	Reference		
Stage of cancer							
Unknown	4	6·0	23	5·4	1·09	0·34, 3·52	0·882
Stage 0	0	0	4	0·9	UD	UD	
Stage 1	12	17·9	63	14·8	0·99	0·45, 2·19	0·995
Stage 2	10	14·9	121	28·3	2·30	1·02, 5·17	0·044
Stage 3	22	32·8	116	27·2	1·00	0·51, 1·95	0·996
Stage 4	19	28·4	100	23·4	Reference		
Site of cancer							
Lip, oral cavity and pharynx (C00–14)	12	17·6	82	18·7	Reference		
Digestive organs (C15–26)	30	44·1	80	18·3	2·56	1·22, 5·35	0·012
Respiratory organs (C30–39)	2	2·9	6	1·4	2·28	0·41, 12·6	0·346
Bone, cartilage, melanoma (C40–43)	4	5·9	142	32·4	1·95	0·55, 6·92	0·3
Breast (C50)	4	5·9	142	32·4	0·19	0·60, 0·62	0·006
Female genital (C51–58)	13	19·1	100	22·8	0·89	0·38, 2·05	0·782
Haematopoietic (C81–96)	2	2·9	7	1·6	1·95	0·36, 10·52	0·436
Kaposi sarcoma (C46)	1	1·5	7	1·6	0·97	0·11, 8·65	0·983
Type of treatment							
Chemotherapy	36	61	323	80·8	Reference		
Surgery	8	13·6	22	5·5	3·26	1·35, 7·86	0·008
Radiotherapy	14	23·7	53	13·3	2·37	1·19, 4·69	0·013
Hormonal therapy	1	1·7	2	0·5	4·49	0·39, 5·71	0·225

UD, undetermined; ESPEN, European Society of Clinical Nutrition and Metabolism.

* Based on ESPEN diagnostic criterion for malnutrition (BMI < 18·5 kg/m^2^).

The overall weight loss >5 % over 3 months was 18·2 % and low FFMI 43·1 %. Malnutrition based on the combined findings of unintentional weight loss of >5 % over 3 months together with age-specific reduced BMI was 8·6 %, and with sex-specific low FFMI was 11·5 %.

The mean FVS was 14·4 (sd 5·6). BMI was significantly associated with the previous 24 h and 7 d FVS at *P* < 0·001 and *P* < 0·003, respectively. A majority (97 %) of participants with low BMI reported inadequate dietary diversity.

### Prevalence and factors associated with cachexia

The prevalence of cachexia based on the Fearon criteria was 14·1 %. Being male, cigarette smoking, alcohol consumption and radiotherapy treatment were significantly associated with cachexia, as shown in Table 4. Males were 3·99 (95 % CI 2·39–6·67; *P* < 0·001) times more likely to have cachexia compared with females. The odds were higher among cigarette smokers (OR 3·95 (95 % CI 2·29, 6·82); *P* < 0·001), alcohol consumers (OR 1·91 (95 % CI 1·15, 3·17); *P* = 0·012) and patients on radiotherapy treatment (OR 2·63 (95 % CI 1·39, 4·97); *P* = 0·003). Patients with breast cancer were 0·19 (95 % CI 0·07, 0·51, *P* = 0·001) times more likely to have cachexia compared with those with lip, oral cavity and pharynx cancer. Unlike malnutrition, patients <30 years of age were 6·52 (95 % CI 2·45, 17·36; *P* < 0·001) times more likely to have cachexia.

**Table 4.** Sociodemographic and clinical factors associated with cachexia (Numbers and percentages; odds ratios and 95 % confidence intervals)

	With cachexia*		Without cachexia*				
Characteristic	*n*	%	*n*	%	OR	95 % CI	*P*
Overall	72	14·1	440	85·9			
Sex							
Female	32	44·4	335	76·1	Reference		
Male	40	55·6	105	23·9	3·99	2·39, 6·67	<0·001
Age							
<30 years	10	13·9	12	2·7	6·52	2·45, 17·36	<0·001
30–39 years	9	12·5	63	14·3	1·12	0·47, 2·65	0·8
40–49 years	17	23·6	116	26·4	1·15	0·56, 2·35	0·708
50–59 years	19	26·4	116	26·4	1·28	0·63, 2·58	0·488
60 years and above	17	23·6	133	30·2	Reference		
Cigarette smoking							
Yes	28	38·9	61	13·9	3·95	2·29, 6·82	<0·001
No	44	61·1	379	86·1	Reference		
Consuming alcohol							
Yes	33	45·8	135	30·7	1·91	1·15, 3·17	0·012
No	39	54·2	305	69·3	Reference		
Stage of cancer							
Unknown	6	8·5	21	4·9	1·33	0·48, 3·71	0·581
Stage 0	0	0·0	4	0·9	UD		0·999
Stage 1	13	18·3	62	14·5	0·98	0·45, 2·09	0·955
Stage 2	9	12·7	125	29·1	0·34	0·15, 0·77	0·01
Stage 3	22	31·0	119	27·7	0·86	0·45, 1·66	0·659
Stage 4	21	29·6	98	22·8	Reference		
Site of cancer							
Lip, oral cavity and pharynx (C00–14)	17	23·6	77	17·5	Reference		
Digestive organs (C15–26)	27	37·5	84	19·1	1·45	0·73, 2·88	0·28
Respiratory organs (C30–39)	2	2·8	7	1·6	1·29	0·24, 6·78	0·76
Bone, cartilage, melanoma (C40–43)	4	5·6	14	3·2	1·29	0·37, 4·42	0·681
Breast (C50)	6	8·3	141	32·0	0·19	0·07, 0·51	0·001
Female genital (C51–58)	13	18·1	103	23·4	0·57	0·26, 1·24	0·16
Haematopoietic (C81–96)	3	4·2	6	1·4	2·26	0·51, 9·97	0·28
Kaposi sarcoma (C46)	0	0·0	8	1·8	UD	UD	
Type of treatment							
Chemotherapy	41	63·1	323	80·8	Reference		
Surgery	6	9·2	24	6·0	1·97	0·76, 5·10	0·163
Radiotherapy	17	26·2	51	12·8	2·63	1·39, 4·97	0·003
Hormonal therapy	1	1·5	2	0·5	3·94	0·35, 44·40	0·267

UD, undetermined.

* Diagnosis based on Fearon criteria for assessment of cachexia (BMI < 20 kg/m^2^ and any degree of weight loss >2 %).

Prevalence of cachexia based on the local definition of cachexia (>10 % weight loss over 6 months) was 8·5 %.

### Predictors of risk of malnutrition, malnutrition and cachexia

Binary logistic regression using the backward conditional method was performed to examine the effect of independent predictors on the risk of malnutrition, malnutrition and cachexia. Age, cigarette smoking and site of cancer were considered for multivariate analysis. The risk of malnutrition was significantly associated with age <30 years (adjusted OR (AOR) 0·21 (95 % CI 0·07, 0·62); *P* = 0·005), the 30–39 years age group (AOR 0·41 (95 % CI 0·20, 0·84); *P* = 0·015), cigarette smoking (AOR 0·36 (95 % CI 0·20, 0·67); *P* = 0·001) and bone, cartilage and melanoma cancer (AOR 2·95 (95 % CI 1·42, 6·12); *P* = 0·004). Malnutrition was significantly associated with age <30 years (AOR 0·12 (95 % CI 0·03, 0·413); *P* = 0·001), cigarette smoking (AOR 0·31 (95 % CI 0·15, 0·65); *P* = 0·002), digestive organ cancer (AOR 6·79 (95 % CI 1·38, 33·34); *P* = 0·018) and breast cancer (AOR 0·30 (95 % CI 0·13, 0·70); *P* = 0·005). Cachexia was significantly associated with age <30 years (AOR 10·71 (95 % CI 3·16, 36·32); *P* < 0·001) and cigarette smoking (AOR 2·72 (95 % CI 1·23, 6·02); *P* = 0·014). Table 5 shows age, cigarette smoking and site of cancer as predictors of risk and presence of malnutrition, and only age and cigarette smoking for cachexia.

**Table 5.** Predictors of risk of malnutrition, malnutrition and cachexia (Adjusted odds ratios and 95 % confidence intervals)

	Risk of malnutrition*			Malnutrition†			Cachexia‡		
	Adjusted OR	95 % CI	*P*	Adjusted OR	95 % CI	*P*	Adjusted OR	95 % CI	*P*
Age									
<30 years	0·21	0·07, 0·62	0·005	0·12	0·03, 0·41	0·001	10·71	3·16, 36·32	<0·001
30–39 years	0·41	0·20, 0·84	0·015	0·43	0·16, 1·18	0·101	2·58	0·98, 6·79	0·054
40–49 years	0·78	0·41, 1·49	0·453	0·52	0·22, 1·24	0·140	1·30	0·55, 3·10	0·549
50–59 years	0·58	0·31, 1·06	0·078	0·92	0·39, 2·20	0·859	1·66	0·75, 3·67	0·212
60 years and above	Reference		Reference		Reference				
Cigarette smoking									
Yes	0·36	0·20, 0·67	0·001	0·31	0·15, 0·65	0·002	2·72	1·23, 6·02	0·014
No	Reference			Reference			Reference		
Site of cancer									
Lip, oral cavity and pharynx	0·73	0·38, 1·41	0·351	Reference					
Digestive organs	0·79	0·14, 4·42	0·788	6·79	1·38, 33·34	0·018			
Respiratory organs	0·68	0·19, 2·48	0·558	0·33	0·05, 2·06	0·238			
Bone, cartilage, melanoma	2·95	1·42, 6·12	0·004	1·06	0·21, 5·27	0·942			
Breast	1·04	0·52, 2·10	0·906	0·30	0·13, 0·70	0·005			
Female genital	0·83	0·15, 4·73	0·838	0·72	0·26, 1·97	0·516			
Haematopoietic	3·83	0·38, 38·84	0·256	0·39	0·06, 2·73	0·345			
Kaposi sarcoma	Reference			0·75	0·07, 7·94	0·808			

MUST, Malnutrition Universal Screening Tool; ESPEN, European Society of Clinical Nutrition and Metabolism.

* Assessed using the MUST.

† Based on ESPEN diagnostic criterion of BMI < 18·5 kg/m^2^.

‡ Based on Fearon criteria of BMI < 20 kg/m^2^ and any degree of weight loss >2 %.

## Discussion

The present study sought to establish the prevalence and factors associated with malnutrition and cachexia among cancer out-patients in Nairobi, Kenya. A third of the patients were found to be at risk of malnutrition, while 13·4 and 14·1 % had malnutrition and cachexia, respectively. Our findings confirm previous results that show malnutrition and cachexia as common nutritional challenges among cancer patients, and that negatively make an impact on the response to cancer treatment, quality of life and overall prognosis^(^^18^^–^^21^^)^. Malnutrition and cachexia are poor prognostic factors and should be prevented or detected and managed early enough^(^^18^^,^^22^^)^. Those found at risk of malnutrition should therefore be closely monitored to avert progression to frank malnutrition. Mechanisms should also be put in place to identify patients at risk of cachexia for the initiation of preventive interventions^(^^3^^)^.

The leading cancers were breast and cervical cancer in females and prostate and oesophageal cancer in males. Our findings agree with the Global Burden of Disease 2016 report that showed prostate and breast cancer as the leading types of cancer in men and women, respectively^(^^23^^)^. Up to 24 % of males and close to 10 % of females presented with oesophageal cancer that has been found to contribute to some of the highest incidences of malnutrition^(^^21^^)^. This might partly explain the significant association observed between malnutrition and cancer of digestive organs in the present study.

Up to 28·2 % presented with stage III cancer disease. However, the odds of being at risk and having malnutrition were higher among patients with stage II cancer disease. Previous studies have shown that many cancer patients in Africa are diagnosed with advanced cancer and easily become malnourished^(^^24^^,^^25^^)^. In light of the present findings on increased odds of malnutrition in early cancer and the fact that 81 % of the patients do not receive nutrition services, there is need for enhanced early screening that can be achieved by integrating nutrition services across the four levels of health care in Kenya.

Many factors can modify nutritional status in cancer patients including nausea, vomiting, decreased energy intake or oncologic treatments resulting in malnutrition^(^^26^^,^^27^^)^. More than 50 % of the patients reported having poor appetite and feeling fatigued, while 35 % felt nauseated and 28 % reported vomiting. Inadequate dietary diversity was associated with low BMI. Previous studies have pointed out alterations that occur in components of energy expenditure that can contribute to malnutrition if not compensated for by increased energy intake^(^^27^^)^. Thus, a holistic approach is required to address the physical, social, psychological and nutritional needs of cancer out-patients to reduce the consequences of cancer-associated nutritional decline^(^^27^^,^^28^^)^.

BMI is the most common and easily available anthropometric assessment method despite its limitation. The local diagnostic criterion is BMI < 18 kg/m^2^, which is similar to the ESPEN criteria. The low BMI criterion was able to identify a higher proportion of patients with malnutrition compared with the combined finding of unintentional weight loss and either a reduced age-specific BMI or a low sex-specific FFMI criteria also recommended by ESPEN. However, the observed low FFMI of 43·1 % highlights the need to incorporate body composition assessments in nutritional assessments. Body composition is not routinely measured in Kenyan public hospitals despite the morbidity risk associated with increased loss of muscle mass. Given its relevance, assessment of body composition should be supported by building the necessary capacity and infrastructure^(^^29^^–^^36^^)^. On the other hand, the Fearon criteria for diagnosis of cachexia identified 14·1 % cachexia compared with 8·5 % using the local criteria. There is need therefore to evaluate the current local diagnostic criteria for cachexia while being cognizant of the local clinical practice and health system structures.

In this study, malnutrition and cachexia were significantly associated with treatment type and site of cancer. A previous study in Kenya among cervical cancer patients undergoing radiotherapy projected a 2-year survival of <20 %^(^^37^^)^. Cancer management therefore requires a multimodal approach, and the Kenyan Government recommends establishment of ‘multidisciplinary tumour boards’ to consider all aspects of the patients’ conditions^(^^7^^)^. A multidisciplinary team is required to consider the implications beyond patients’ dietary needs to patients’ nutritional and functional state throughout the prolonged course of treatment, and is best started earlier rather than later^(^^20^^,^^35^^,^^38^^)^. The role of nutrition as a therapy that complements basic treatment and improves treatment outcomes needs to be appreciated^(^^22^^,^^38^^,^^39^^)^. Inclusion of nutritionists in mainstream oncology practice has the potential to improve early detection and screening of malnutrition, access to nutrition services and communication for improved patient management^(^^22^^,^^40^^)^. Awareness and consideration of nutritional issues among oncologists and other related health disciplines are also vital to the success of nutrition support in cancer care^(^^24^^,^^36^^)^. In settings with limited nutritionists/dietitians, provision of protocols for screening can enable task shifting that has been shown to be effective and affordable in improving access to healthcare^(^^41^^)^.

Findings on sociodemographic and behavioural factors add insights to the likely influence of social factors in the management of cancer. Disease-aggravating factors such as cigarette smoking and alcohol consumption were prevalent and significantly associated with the presence of malnutrition. Cigarette smoking was a predictor of cachexia. Public sensitisation on the ill-effects of such risk factors and raising the suspicion index among healthcare workers is important. The Global Burden of Disease 2016 report^(^^23^^)^ showed that mortality due to all cancers is largely located on the African continent and expected to increase due to the epidemiological transition. Continued research and surveillance therefore remain critical. Further research is needed to understand the nutrition requirements in various care settings, and explore novel cancer preventive and control strategies^(^^19^^,^^38^^)^. The emergence of population-based cancer registries in East Africa should be supported, and so should prioritisation of cancer control programmes within the health-care systems^(^^19^^,^^42^^–^^44^^)^.

### Conclusions

Malnutrition and cachexia remain a challenge among cancer out-patients in Nairobi, Kenya, yet the provision of nutritional services remains low. The use of the MUST as a screening tool at the first point of care has the potential to identify patients at risk of malnutrition and who are likely to benefit from appropriate interventions for better health outcomes. The local diagnostic criteria for cachexia should be reassessed to ensure correct identification of patients at risk of cachexia for initiation of appropriate care. There is need for inclusion of body composition assessments in Kenyan public hospitals for refined nutritional assessments that will in turn inform patient management plans. Age is a common predictor of malnutrition and cachexia in addition to site of cancer for malnutrition and cigarette smoking for cachexia. Tailored education and sensitisation campaigns should therefore be encouraged and multidisciplinary partnerships formed to provide holistic cancer care.

### Limitations

The study did not measure inflammatory markers or ascertain the aetiology of malnutrition observed. The contribution of inflammation and other catabolic drivers to the development and progression of cachexia was also not assessed. This was partly due to the design of the study and the choice of out-patients in whom tests for inflammatory markers are not routinely done in the selected study facilities.
